# Overexpression of LASP-1 mediates migration and proliferation of human ovarian cancer cells and influences zyxin localisation

**DOI:** 10.1038/sj.bjc.6603545

**Published:** 2007-01-09

**Authors:** T G P Grunewald, U Kammerer, C Winkler, D Schindler, A Sickmann, A Honig, E Butt

**Affiliations:** 1 Institute of Clinical Biochemistry and Pathobiochemistry, University of Wurzburg, Grombuehlstr. 12, D-97080 Wurzburg, Germany; 2 Department of Obstetrics and Gynecology, University of Wurzburg, Josef-Schneider-Str. 4, D-97080 Wurzburg, Germany; 3 Protein Mass Spectrometry and Functional Proteomics Group, Rudolf-Virchow-Center for Experimental Biomedicine, Versbacher Straße 9, 97078 Wurzburg, Germany; 4 Department of Human Genetics, University of Wurzburg, Biozentrum am Hubland, D-97074 Wurzburg, Germany

**Keywords:** LASP-1, ovarian cancer, zyxin, 14-3-3, SKOV-3, migration

## Abstract

LIM and SH3 protein 1 (LASP-1), initially identified from human breast cancer, is a specific focal adhesion protein involved in cell proliferation and migration. In the present work, we analysed the effect of LASP-1 on biology and function of human ovarian cancer cell line SKOV-3 using small interfering RNA technique (siRNA).Transfection with LASP-1-specific siRNA resulted in a reduced protein level of LASP-1 in SKOV-3 cells. The siRNA-treated cells were arrested in G_2_/M phase of the cell cycle and proliferation of the tumour cells was suppressed by 60–90% corresponding to around 70% of the cells being transfected successfully as seen by immunofluorescence. Moreover, transfected tumour cells showed a 40% reduced migration. LASP-1 silencing is accompanied by a reduced binding of the LASP-1-binding partner zyxin to focal contacts without changes in actin stress fibre and microtubule organisation or focal adhesion morphology as observed by immunofluorescence. In contrast, silencing of zyxin is not influencing cell migration and had neither influence on LASP-1 expression nor actin cytoskeleton and focal contact morphology suggesting that LASP-1 is necessary and sufficient for recruiting zyxin to focal contacts.The data provide evidence for an essential role of LASP-1 in tumour cell growth and migration, possibly through influencing zyxin localization.

Ovarian cancer is the sixth most common cancer among women worldwide, with estimated 190 000 new cases and 114 000 deaths caused through this neoplasm each year (Parkin *et al*, 2001). Epithelial ovarian cancer represents 90–95% of all ovarian tumours (Auersperg *et al*, 2001; Quirk and Natarajan, 2005), which is detected in 60.6% cases in advanced stages of disease due to unspecific or absent symptoms in early stages. Although tremendous efforts have been undertaken to improve the therapeutic outcome, ovarian cancer still remains the most lethal malignoma among gynaecological tumours of women in the western world, given that only 35% of ovarian cancer patients show a 5-year survival (Legge *et al*, 2005).

About 90% of all epithelial ovarian tumours are sporadic and are diagnosed in women without germline mutations in known susceptibility loci. However, the remaining cases are heritable. Data from several international studies suggest that patients with a loss of heterozygosity of the BRCA1 and BRCA2 genes, which are located on 17q21 and found in hereditary forms of breast cancer, have a 6–61-fold increased lifetime risk of ovarian cancer compared with general population rates (Antoniou *et al*, 2003).

The Lim and SH3 domain protein LASP-1 was initially identified from a cDNA library of breast cancer metastases. The gene was also mapped to human chromosome 17q21 in a region that is altered in 20–30% of human breast cancers (Tomasetto *et al*, 1995a, 1995b), suggesting that it could play a role in tumour development and metastasis of breast and ovarian cancer.

Human LASP-1 encodes a membrane-associated protein of 261 amino acids containing an N-terminal LIM domain, followed by two actin-binding sites and a C-terminal src homology SH3 domain. The actin-binding domains in the core of the LASP-1 protein mediate an interaction between LASP-1 and actin at cell membrane extensions, but not along the actin stress fibres (Schreiber *et al*, 1998; Chew *et al*, 2002; Butt *et al*, 2003; Keicher *et al* 2004; Nakagawa *et al*, 2006). The exact cellular function of LASP-1 is still not known, but the protein has previously been reported to localise within multiple sites of dynamic actin assembly such as focal contacts, focal adhesions, lamellipodia, membrane ruffles and pseudopodia (Tomasetto *et al*, 1995a; Chew *et al*, 1998, 2000, 2002; Lin *et al*, 2004).

The SH3 domain at the C-terminus is involved in protein–protein interactions through binding to proline-rich sequences, specifically with zyxin, pallidin, lipoma preferred partner (LPP) and vasodilator-stimulated phosphoprotein (VASP) (Keicher *et al*, 2004; Li *et al*, 2004; Rachlin and Otey, 2006).

Moreover, recent data showed that LASP-1 specifically interacts via its nebulin-like repeats with Krp1, a focal adhesion protein involved in cell migration. Mutation analysis of LASP-1 demonstrates that its SH3 domain is necessary for pseudopodial extension and invasion (Spence *et al*, 2006). Thus the protein–protein interactions mediated by the LIM and SH3 domains can be regarded as scaffolds for the formation of complexes of higher order.

LASP-1 is a cAMP- and cGMP-dependent protein kinase substrate with a specific phosphorylation site on serine 146 (Butt *et al*, 2003). In rabbit parietal cells, elevation of intracellular cAMP by forskolin induced a partial translocation of LASP-1 to the apically directed F-actin-rich intracellular canaliculus, which is the site of active HCl secretion (Chew *et al*, 1998, 2000). Beyond this, phosphorylation on serine 146 resulted in translocation of the protein from the membrane to the cytosol and was followed by reduced cell migration (Butt *et al*, 2003). It has also been shown that the SH3 domain of LASP-1 interacts with the N-terminus of Ableson tyrosine kinase and that phosphorylation of LASP-1 at tyrosine 171 is associated with the loss of LASP-1 from focal adhesions and the initiation of cell death, but without changes in dynamic of migratory processes (Lin *et al*, 2004).

In addition, LASP-1 expression has been reported to be increased in metastatic breast cancer, suggesting that overexpression of LASP-1 may be involved in the migratory process of these cells (Tomasetto *et al*, 1995a). Surprisingly, both increase and depletion of LASP-1 in COS-7, HEK293 and MCF-7 cells inhibited basal and growth factor-stimulated cell migration (Lin *et al*, 2004).

Interestingly, recent work has shown, that knock-down of LASP-1 in metastatic breast cancer cell lines BT-20 and MCF-7 results in a strong inhibition of proliferation and migration and leads to a reduction of zyxin at focal contacts through absence of LASP-1 (Grunewald *et al*, 2006).

In this study we demonstrate that LASP-1 is highly overexpressed in ovarian cancer tissue and metastatic ovarian cancer cell lines. Silencing of the LASP-1 gene by RNA interference in the ovarian cancer cell line SKOV-3 reduced cell proliferation and cell migration *in vitro* without influencing the actin cytoskeleton, microtubule polymerisation and focal adhesion morphology. Furthermore, the knock-down of LASP-1 severely affected zyxin localisation.

## MATERIALS AND METHODS

### Tissue samples

The studies were performed with approval of the Ethics Committee of the University of Wurzburg. Tissue samples of 26 archival cases each of serous epithelial ovarian carcinomas with and without invasive components (obtained from the Department of Pathology of the University of Wurzburg and reviewed by a pathologist to confirm the diagnosis), as well as two samples of ascitic fluid containing ovarian cancer cells of women with metastatic ovarian cancer were analysed.

### Immunohistochemistry

For immunohistochemical staining procedures, endogenous peroxidase was blocked by incubation in 0.1% hydrogen peroxide in PBS for 5 min. The slides were then incubated with the polyclonal anti-LASP-1 antibody (Butt *et al*, 2003) diluted 1 : 1000 in ‘antibody diluent’ (DAKO, Hamburg, Germany), followed by the EnVision/rabbit detection system (DAKO). Histogreen (Linaris, Wertheim, Germany) was used as the chromogen and cells were counterstained with haematoxylin (Sigma, Deisenhofen, Germany).

### Cell culture conditions

Cell lines (SKOV-3, OAW-42 and PA-1) were obtained from the Cell Line Services (Heidelberg, Germany) and grown at 1 × 10^5^ cells ml^−1^in a plastic cell culture flask in a humidified incubator at 37°C under 5% CO_2_ atmosphere in RPMI 1640 medium (PAA, Linz, Austria) containing 10% heat-inactivated foetal bovine serum (PAA) and 1% streptomycin/ampicillin (Invitrogen, Karlsruhe, Germany). For primary tumour cell culture, effusions (20–500 ml) were centrifuged, cell pellets washed twice in PBS (Biochrom, Berlin, Germany),resuspended in RPMI 1640 medium supplemented as described and then seeded on the bottom of a cell culture flask. After 1 h, all nonadherent (mostly leucocytes) cells were washed away and adherent tumour cells cultured in RPMI 1640/10%FCS/streptomycin/ampillicin. Contaminating fibroblasts were deleted by trypsin treatment every other day and the remaining tumour cell monolayer was cultured until homogeneous morphology of the cells (passage 3–4) was reached. Cells were checked routinely and found to be free of contamination by bacteria or fungi.

### small interfering RNA preparation and transfection

Expression of human LASP-1 was knocked down with siRNA duplexes targeting the sequence 5′-AAG GTG AAC TGT CTG GAT AAG-3′ (bases 49–69), 5′-CUUAUCCAGACAGUUCACCdTdT-3′. The control siRNA 5′-AGAGAUGUAGUCGCUCGCUdTdT-3′ targeting no known mRNA sequence was used as a control. Both siRNAs were obtained from Dharmacon RNA Technologies (Lafayette, CO, USA). The siControl nontargeting siRNA from Dharmacon could not be used in our cell system owing to toxic effects. A BLAST search against the complete human and murine genome verified that the selected sequences were specific for the respective target gene.

Cells in the exponential phase of growth were plated in six-well plates at a density of 0.5 × 10^5^ cells/well, grown for 24 h and transfected with 1 *μ*g (60 nM) siRNA in reduced serum medium OPTI-MEM-I (Gibco, Paisley, UK) at 30–50% confluence. For the formation of the siRNA–lipid complexes, 3 *μ*l siRNA stock solution (20 *μ*M) was diluted in 100 *μ*l OPTI-MEM, mixed with 3 *μ*l Metafectene (Biontex, Munich, Germany) in 100 *μ*l OPTI-MEM and incubated at room temperature for 17 min. Subsequently, the culture medium was removed and replaced by 1 ml OPTI-MEM-I and the siRNA–lipid complexes (1.2 ml total volume). After 4 h incubation at 37°C, 1.2 ml of cell culture medium with 20% foetal bovine serum was added, and incubation was continued for 36–56 h. For control cells, Metafectene alone (MOCK-transfection) and/or 1 *μ*g scrambled control-siRNA were used.

Silencing of zyxin was achieved with Hs_ZYX_1_HP validated siRNA at a final concentration of 10 nM using HiPerfect transfection reagent (Qiagen, Hilden, Germany) according to the manufacturer's directions. For zyxin control experiments, nonsilencing control siRNA, Alexa Fluor 488 labelled, provided by Quiagen, was used.

At least three independent experiments were performed for each cell line, and representative results are shown.

### Cell proliferation assay

For proliferation assay, cells were transfected as described. At the time points indicated, cells were trypsinised and cell numbers were determined by a Coulter counter (Beckman, Fullerton, CA, USA). Experiments were performed in triplicate for each time point. Cell viability was evaluated by counting trypan blue-positive and -negative cells under a phase-contrast microscope (Zeiss Axiovert, Aalen, Germany).

### Western blot analysis

For Western blotting, cells were prepared by lysing in Laemmli sample buffer and equal amounts of protein, according to the cell count, were resolved by 12% SDS–PAGE. After blotting on nitrocellulose membrane and blocking with 3% nonfat dry milk in 10 mM Tris, pH 7.5, 100 mM NaCl and 0.1% (w/v) Tween 20, the membrane was first incubated with the antibodies raised against LASP-1 (1 : 10 000) (Butt *et al*, 2003), caspase-3 (1 : 1000) (New England Biolabs, Frankfurt, Germany) or mouse zyxin hybridoma supernatant (1 : 100; kind gift from Dr J Wehland, GBF Braunschweig, Germany; Rottner *et al*, 2000), followed by incubation with horseradish peroxidase-coupled goat anti-rabbit IgG or goat anti mouse IgG (Biorad, Munich, Germany), diluted 1 : 5000 and detection by ECL or ECL plus (Amersham Biosciences, Freiburg, Germany). Protein bands were visualised by autoradiography. Quantification of the signals was carried out by densitometry using the Odyssey system (Li-Cor, Bad Homburg, Germany).

### FACS

For cell cycle analysis, SKOV-3 cells were harvested 48 h after LASP-1 siRNA transfection. The cells were pelleted and stained with DAPI (Sigma) at a final concentration of 2 *μ*g ml^−1^ in permeabilisation buffer containing 0.1 M Tris—HCl, pH 7.4, 0.154 M NaCl, 0.5 mM MgCl_2_, 1 mM CaCl_2_, 0.1% NP-40 and 0.2% BSA in ddH_2_O for 30 min at 4°C in the dark. Bivariate flow histograms were recorded on an analytical, dual-laser-equipped cytometer (LSR1, Becton Dickinson Biosciences, Heidelberg, Germany) using UV excitation. Resulting cell cycle distributions were quantified with the MPLUS AV software (Phoenix Flow Systems, San Diego, CA, USA). For technical details, see Schindler and Hoehn (1999).

### Two-dimensional-gelelectrophoreses and mass spectrometry

Isoelectric focusing for two-dimensional (2D) gel electrophoresis was performed using the Protean IEF cell from Biorad (Munich, Germany) according to the instructions of the manufacturer. The SKOV-3 homogenate (about 200 *μ*g protein) was solubilised for 15 min by sonication in 320 *μ*l lysis buffer containing 7 M urea, 2 M thiourea, 4% (w/v) CHAPS, 15 mM DTT (electrophoresis grade), 0.5% carrier ampholytes, pH 3–10. Pellet homogenate was loaded on a 17-cm immobilised IPG strip, pH 3–10 and resolved overnight at 50 V. Focussing was carried out for 1 h at 250 V, 1 h at 500 V and 15 h at 7000 V. After equilibration in 50 mM Tris, pH 8.9, 6 M urea, 30% (w/v) glycerol and 2% (w/v) SDS, gels were immediately applied to a vertical 10% SDS gel without a stacking gel. Electrophoresis was carried out at 8°C with a constant current of 40 mA per gel. Proteins were visualised by Coomassie Brilliant Blue R-250 (Sigma) staining.

Gel pieces were washed two times alternating with 50 mM ammonium hydrogen carbonate buffer and 25 mM ammonium hydrogen carbonate buffer with 50% acetonitrile. Proteins were reduced with 10 mM DTT for 30 min at 56°C and subsequently alkylated by incubation with 20 mM iodoacetamide at room temperature for 30 min. Again samples were washed as described before. Gel pieces were shrunken in a SpeedVac (Thermo Electron, Dreieich, Germany) and rehydrated with 12.5 ng of trypsin in 50 mM ammonium hydrogen carbonate buffer. Digestion was performed by incubation at 37°C overnight. The resulting peptides were extracted by application of 15 *μ*l of 5% formic acid for 10 min.

Separation of complex peptide mixtures was achieved by using reversed-phase chromatography. For nano-LC-ESI-MS/MS experiments, a setup consisting of an autosampler (Famos, Dionex, Idstein, Germany) and precolumn concentration (Switchos, Dionex) before nano-LC separation (Ultimate, Dionex) was used. Precolumns (300-*μ*m inner diameter × 1-mm length) and separation columns (75 *μ*m inner diameter × 150-mm length, C18 PepMapTM) were purchased from Dionex. Gradient elution was performed using a linear gradient from 5 to 50% solvent B (84% acetonitrile, 0.1% formic acid) during a period of 2 h. Solvent A was 0.1% formic acid in water. Separation was followed by rinsing the column with 95% B for 5 min before equilibration to 5% solvent B before the next separation cycle.

Peptides were directly eluted into an ESI mass spectrometer. For mass spectrometric analysis, an ESI linear ion trap LTQ (Thermo Electron, Dreieich, Germany), using distal-coated fused silica tips (New Objective, Woburn, MA, USA), spray voltage was set around 1800 V. A survey scan (m/z 350–2000) was followed by five MS/MS scans fragmenting the five most intensive peptide signals.

Mass spectra were transformed into peak lists in dta or mgf format using the wo in-house software solution raw2dta (Boehm *et al*, 2004). Generated data were processed in parallel with the search algorithms SequestTM, Version 27 (Yates *et al*, 1995) and MascotTM, Version 2.1.6 (Perkins *et al*, 1999). For sequence alignment, the swissprot database from October 2005 was used. As fixed modification, carbamidomethylation of cysteine residues was used, and as variable modification oxidation of methionine residues was selected. As filter criteria for Sequest we accepted in the first instance only positive peptide hits with a minimum cross-correlation factor of 2.5, a CN value of 0.25, and a preliminary ranking of one. For the Mascot algorithm the minimum score was set to 40 for each peptide. Only protein hits that were identified with these parameters by both algorithms and had at minimum two identified peptides were accepted. Additionally, all significant hits were revised manually.

### Immunfluorescence

For immunfluorescence microscopy, cells were grown on glass chamber slides, fixed in 4% (w/v) paraformaldehyde in PBS, permeabilised with 0.1% (w/v) Triton X-100 in PBS and then stained with affinity-purified LASP-1 antibody (1 : 2000, 1 h), followed by secondary Cy3-labelled anti-rabbit antibody (1 : 500, 30 min) (Dianova, Hamburg, Germany) or mouse zyxin hybridoma supernatant (1 : 10, 2 h), followed by secondary Cy2 labelled goat-anti mouse antibody (1 : 500, 1 h) (Dianova). Oregon green phalloidin (Molecular Probes, Leiden, The Netherlands) was used for actin staining. Tubulin was stained with an anti-*α*-tubulin antibody (3 *μ*g ml^−1^) (Calbiochem, Darmstadt, Germany). DNA was counterstained with DAPI (1 : 2500) (Calbiochem) for 2 min.

### Migration experiments

Cells were cultured in medium in 25 cm^2^ flasks to approximately 30–40% confluence and transfected with 10 nM zyxin siRNA or 30 nM LASP-1 siRNA (Keicher *et al*, 2004) using 10 *μ*l Metafectene. After 48 h incubation and overnight starving, 1 × 10^5^ cells in 100 *μ*l incubation medium (with 1 mM MgCl_2_) were seeded in the upper chamber of BSA-coated 8 *μ*M pore size transwell Boyden chambers (Corning star, Cambridge, MA, USA). Cells were allowed to migrate through the porous membrane for 4 h at 37°C. Cells remaining at the upper surface were completely removed using a cotton carrier. The lower surfaces of the membranes were then stained in a solution of 1% (w/v) crystal violet in 2% ethanol for 30 s and rinsed afterwards in distilled water. Cell-associated crystal violet was extracted by incubation in 10% acetic acid for 20 min and measured at 595 nm absorbance.

## RESULTS

### LASP-1 is overexpressed in ovarian cancer tissue

**Figure 1:**
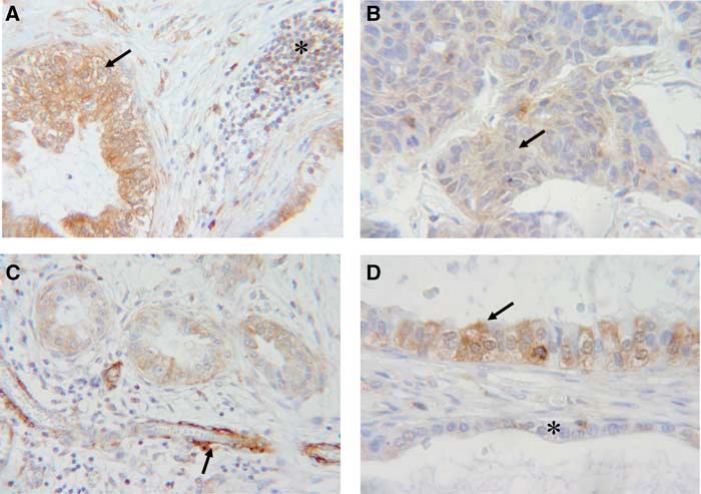
Immunohistochemical staining of cancerous ovarian tissue. LASP-1 was detected using anti-LASP-1 rabbit polyclonal antibody in paraffin-embedded tissue samples. (**A**) Ovarian cancer tissue with two cystic structures containing malignant cells, which are strongly LASP-1 positive (arrow), and an LASP-1-negative lymphocytic inflammation (star). (**B**) Infiltrating tumour cells displaying medium LASP-1 expression (arrow). (**C**) Three ductus with malignant epithelial cells and medium LASP-1 expression and a blood vessel in longitudinal cut with vascular smooth muscle cells showing strong LASP-1 positivity (arrow). (**D**) Two epithelial strata separated through connective tissue. The arrow indicates malignant LASP-1-positive and the star benign LASP-1-negative epithelial cells. LASP-1^+^ cells are stained in brown (DAB). All sections were counterstained with haematoxylin.

To assess the role of LASP-1 in ovarian cancer, we examined its expression in 26 ovarian cancer samples from different patients with or without invasive components. Immunohistochemistry clearly allowed to localise LASP-1 expression in 14 out of 26 malignant ovarian tissues (53.8%). Strong immunoreactivity was observed in nine cases (Figure 1A), whereas five probes showed a medium to low LASP-1 expression (Figure 1B) and 12 specimens (46.2%) were considered to be LASP-1 negative.

Normal benign epithelial cells were LASP-1-negative in all ovarian tissues even when malignant epithelial cancer cells close to these normal epithelial cells displayed a strong positivity for LASP-1 (Figure 1D).

In analogy to previous findings in myoepithelial cells of human breast tissue (Grunewald *et al*, 2006), a massive overexpression in vascular smooth muscle cells could be observed (Figure 1C).

### LASP-1 is strongly expressed in ovarian cancer cell lines

In order to study the significance of LASP-1 overexpression in ovarian cancer, we tested three ovarian cancer cell lines (SKOV-3, OAV-42 and PA-1) as well as two primary cell cultures derived from ascitic fluid of patients with peritoneal metastatic ovarian cancer for LASP-1 expression. Loading was standardised to 3 × 10^5^ cells per slot and controlled by *β*-actin loading control signal intensity.

**Figure 2:**
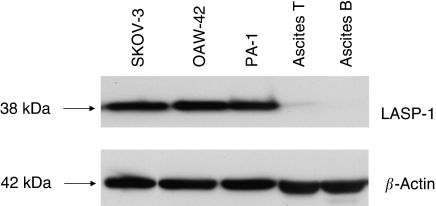
Western blot of different ovarian cancer cell lines. A total of 200 000 cells from established ovarian cancer cell lines (SKOV-3, OAW-42 and PA1), as well as primary cells derived from ascitic fluid of two patients (ascitis T and B) with peritoneal metastatic ovarian cancer, were analysed for LASP-1 expression by Western blot. Loading was controlled by *β*-actin Western blot.

Only the three ovarian cancer cell lines showed a high LASP-1 signal, whereas the two primary cell lines were LASP-1 negative (Figure 2). Interestingly, the solid primary ovarian cancer tissue preparations of these two patients showed intensive LASP-1 staining (data not shown).

We chose SKOV-3 cells as a cellular model for ovarian cancer because in these cells the BRCA1 and BRCA2 genes, which are located next to the LASP-1 gene on chromosome 17q21, are upregulated (Rauh-Adelmann *et al*, 2000).

### Silencing of LASP-1 in SKOV-3 cells inhibits proliferation *in vitro*

**Figure 3:**
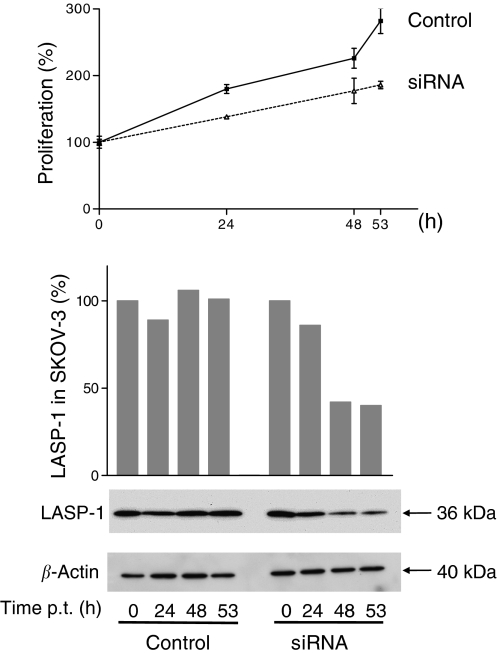
Silencing of LASP-1 in SKOV-3 cells inhibits proliferation. A total of 40 000 cells of the SKOV-3 cells were plated and allowed to grow for 24 h (up to 40% confluence). Small interfering RNA LASP-1 was transfected into cells in a concentration of 60 nM. Cells were harvested after 0, 24, 44 and 53 h of siRNA treatment. Control cells were treated with control siRNA. Upper panel: treatment with siRNA LASP-1 impairs SKOV-3 cell proliferation. After the indicated periods of time, the cells were harvested, and their total number was determined using a Coulter counter. Lower panel: Densitometric quantification and Western blot analysis of LASP-1 expression standardised to *β*-actin at the corresponding time points shows a reduction of LASP-1 expression of about 60% 44 h after transfection with siRNA LASP-1.

**Figure 4:**
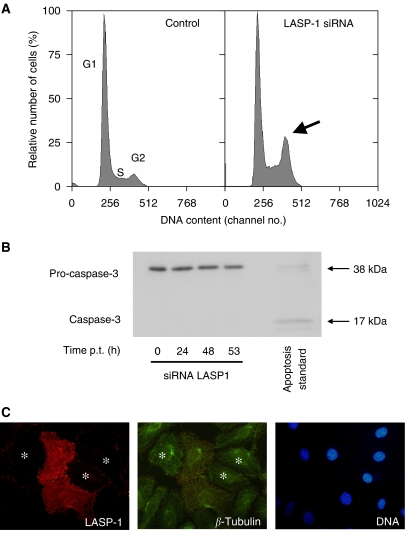
LASP-1 siRNA treatment of SKOV-3 cells induces G_2_ phase accumulation without triggering apoptosis. (**A**) SKOV-3 cells were transfected with siRNA LASP-1 and transfection reagent Metafectene or treated only with Metafectene (MOCK transfection). After 48 h, the cells were harvested and prepared for flow cytometric cell cycle analysis. LIM and SH3 protein 1 (LASP-1) siRNA-treated cells show G_2_ phase accumulation as opposed to the same cells with MOCK transfection. (**B**) Several hours after siRNA LASP-1 treatment, cells were harvested and prepared for Western blot analysis using an anti-caspase-3 antibody, the antibody recognises both the nonactive pro-caspase-3 (38 kDa) and the active cleaved caspase-3 protein (17 kDa). No active caspase-3 could be detected. (**C**) Immunfluorescence of LASP-1 (red), *α*-tubulin (green) and DNA (blue) of siRNA LASP-1-transfected (_^*^_) and nontransfected SKOV-3 cells.

To investigate the function of LASP-1 in the ovarian cancer cell line SKOV-3, we performed a knock-down of the gene using the powerful RNAi technique. The effect of siRNA transfection on the expression of LASP-1 was followed by Western blot analysis 0, 24, 48 and 53 h after transfection. The amount of LASP-1 protein, standardised to *β*-actin, was reduced up to 58% after 48 h (Figure 3, lower panel) compared with the control siRNA-transfected control cells. This corresponds to around 70% of the cells being transfected successfully as seen by immunofluorescence. Parallel to the protein knock-down, we observed a slower proliferation rate in LASP-1 siRNA-transfected cells compared with control cells (Figure 3, upper panel). The viability of cells was similar in both cultures as trypan blue staining detected no more than 5–8% dead cells in all experiments. To test whether the decreased proliferation could be due to apoptosis, we carried out a Western blot analysis with an anti-caspase-3 antibody. The antibody recognises both the non-active pro-caspase-3 (38 kDa) and the active cleaved caspase-3 protein (17 kDa). As shown in Figure 4B, treatment of SKOV-3 cells with the LASP-1 siRNA duplex produced no active caspase-3, indicating that apoptosis might not explain the reduced proliferation.

### Downregulation of LASP-1 induces G2 phase accumulation in SKOV-3 cells

We next analysed cell cycle distributions of siRNA-treated SKOV-3 cells using flow cytometry. After incubation with LASP-1 siRNA for 48 h, the proportion of cells accumulating in the G_2_ phase amounted to 19.4% (Figure 4A), whereas the same cells treated with Metafectene alone (MOCK transfection) as a control had only 6.7% G_2_ phase proportion. Conversely, the G_1_ fraction decreased from 73.4% in MOCK-treated cells to 48% in LASP-1-silenced SKOV-3 cells. The S phase fraction for siRNA LASP-1-treated cells was 32.6% and for MOCK transfection 19.9%, respectively. Similar results were obtained in three independent experiments indicating that in LASP-1-silenced SKOV-3 cells mitotic progression cannot proceed normally. However, immunfluorescence staining of *α*-tubulin and DNA in the cells reveald no reduced tubulin polymerisation in the LASP-1-silenced cells arrested in G_2_/M phase (Figure 4C).

### Knock-down of LASP-1 results in protein changes of glycolytic metabolism and cell cycle regulation

**Figure 5:**
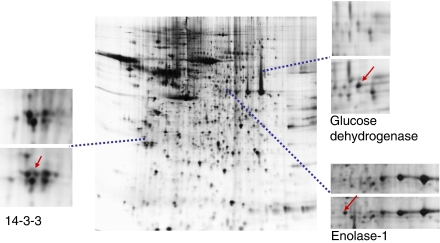
Two-dimensional gel of SKOV-3 proteins after LASP-1 silencing. Sections are showing differences between cellular proteins from control (upper panel) and LASP-1 knock-down (lower panel) SKOV-3 cells. Proteins were separated on nonlinear IPG-strips, pH 3–10. Two dimensional gels were stained with Coomassie blue. Proteins were identified following tryptic digestion and analysis of the resulting peptides by ESI-MS/MS.

Under LASP-1 knock-down conditions it might be necessary for the maintenance of cellular steady state to upregulate alternative proteins to overcome functionally the loss of LASP-1. We used 2D gel electrophoreses to resolve the homogenate of SKOV-3 cells before and after LASP-1 silencing. Subtractive analysis of the two gels showed a high degree of similarity, however, at least five proteins have been identified to become up-, or respectively downregulated by LASP-1 silencing in three independent experiments: pyruvate kinase (up), enolase-1 (down), glucose dehydrogenase (down), 14-3-3 (up) and heat-shock protein (Hsp) 27 (up) (Figure 5).

### Silencing of LASP-1 results in reduced zyxin binding to focal adhesions

**Figure 6:**
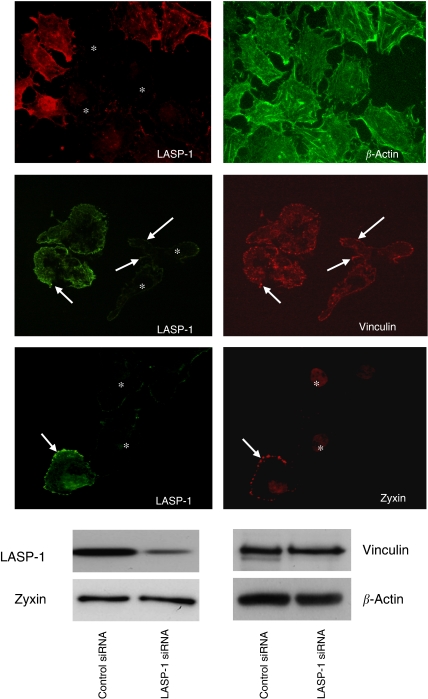
LIM and SH3 protein 1 (LASP-1) is required for zyxin localisation at focal adhesions. Immunfluorescent images of LASP-1, zyxin, vinculin and *β*-actin in siRNA LASP-1-treated SKOV-3 cells. Focal adhesions are marked with white arrows. Positions of downregulated cells are marked with stars. Western blot (WB) analysis to assess LASP-1 and zyxin levels in the LASP-1 siRNA and control siRNA-treated SKOV-3 cells were performed from the corresponding immunofluorescent cell extracts.

LASP-1 has previously been shown to localise to sites of cell adhesion and to interact with zyxin and actin (Chew *et al*, 2002; Li *et al*, 2004). To assess whether silencing of LASP-1 affects these binding partners, siRNA-treated SKOV-3 cells were stained with phalloidin green against actin or mouse anti-zyxin hybridoma supernatant. In LASP-1 siRNA-transfected cells, zyxin was absent from focal adhesions, whereas the cellular level of zyxin remained unchanged as confirmed by Western blot analysis (Figure 6). However, absence of zyxin from focal contacts did not lead to changes in focal adhesion morphology as visualized by vinculin staining (Figure 6). Likewise, actin filament assembly was not disturbed (Figure 6), despite less actin bundles, a blurred network of shorter filaments and some F-actin aggregates are typical for highly metastatic cancer cell lines (Liu *et al*, 2004).

### Silencing of zyxin does not change LASP-1 localisation or focal adhesion morphology

**Figure 7:**
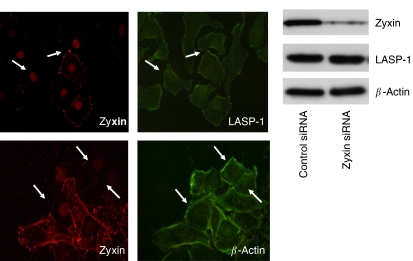
Zyxin silencing is not influencing actin and LASP-1 localisation. Immunoflourescent images of SKOV-3 cells transfected with siRNA zyxin and stained with antibodies against LASP-1 and *β*-actin. Shown are representative sections of a mixed population of both, zyxin downregulated cells and nontransfected cells, demonstrating-no changes in actin and LASP-1 distribution of cells lacking zyxin.

As knock-down of LASP-1 is altering zyxin localisation, we further assessed the interaction of both proteins in a reverse experiment by knocking down zyxin. Transfection of SKOV-3 with zyxin-specific siRNA dramatically reduced zyxin expression down to 10–20%, while the cellular level of LASP-1 and *β*-actin remained unchanged as confirmed by Western blot analysis (Figure 7). Immunofluorescence of the zyxin-silenced cells illustrated lack of zyxin at the focal adhesions without altering the position of LASP-1 (Figure 7), suggesting that LASP-1 is necessary for the positioning and recruiting of zyxin to focal adhesions. Other focal adhesion proteins, for example, *β*-actin (Figure 7) and vinculin (data not shown), were unaffected by the zyxin knock-down.

### Silencing of LASP-1, but not of zyxin, decreases cell migration

**Figure 8:**
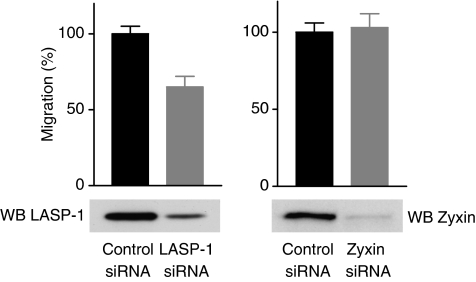
LIM and SH3 protein 1 is necessary for cell migration. SKOV-3 cells were transfected with LASP-1 siRNA, zyxin siRNA or control siRNA. Migration was measured over 4 h in a Transwell® cell culture chamber. At least four chambers from three different experiments were analysed (*P*-values significantly different from that of Control by *t*-test; *P*<0.001). Each bar represents the mean±s.d. Corresponding Western blots of control cells and LASP-1 siRNA-transfected or zyxin siRNA-transfected cells are shown in the lower panel.

Although the exact function of LASP-1 is not known, recent results suggest an important role for this protein in cell adhesion and migration (Butt *et al*, 2003; Lin *et al*, 2004; Grunewald *et al*, 2006; Nakagawa *et al*, 2006). To directly examine the relevance of LASP-1 for cell motility we performed migration experiments in a modified Boyden chamber with SKOV-3 cells either transfected with LASP-1 siRNA or zyxin siRNA to downregulate the respective protein. Cells were seeded in the upper chamber of a transwell polycarbonate membrane. After 4 h, those cells that had migrated through the porous membrane were counted. Depletion of LASP-1 in SKOV-3 cells strongly reduced cell migration, while zyxin knock-down had no influence on cell migration (Figure 8) suggesting that LASP-1 acts as a positive regulator for cell motility.

## DISCUSSION

Cell migration and controlled assembly and disassembly of focal adhesions are highly integrated multistep processes and a central feature in the molecular pathology of cancer (Ridley *et al*, 2003). To date, more than 50 different adhesion proteins that regulate the rate and organisation of actin polymerisation and focal adhesion turnover in protrusion have been identified.

In earlier publications, overexpression of LASP-1 mRNA in metastatic lymph nodes derived from breast cancer patients, as well as the co-amplification of the gene together with HER-2/neu (c-erbB2) were demonstrated (Chew *et al*, 1998; Legge *et al*, 2005). Two additional observations underscore the importance of LASP-1 in cancer. First, altered expression of LASP-1 is associated with the MLL gene in acute myeloid leukaemia (Strehl *et al*, 2003). Second, recent studies have shown LASP-1 to be transcriptionally upregulated in response to the morphogen Sonic Hedgehog (Ingram *et al*, 2002).

Consistent with these data, we just recently described the overexpression of LASP-1 to very high levels in breast carcinomas and lymph node metastases (Grunewald *et al*, 2006). The functional significance of LASP-1 in cancer metastasis is further supported by the presented data showing high LASP-1 expression in ovarian cancer tissue and reduced cell migration in ovarian cancer cells depleted of LASP-1. The absence of LASP-1 in cultures of primary ovarian cancer cells in contrast to established cell lines may reflect a downregulation of LASP-1 in the nonmigratory floating ascites cells which will be reverted after several passages of adherent cell culture. Comparable observations are published for LASP-1 in human mesenchymal stem cells showing an upregulation of the protein during later passages (Sun *et al*, 2006).

During LASP-1 silencing we observed reduced cell cycle progression and an induced G_2_/M phase accumulation of the cells without disrupted normal mitotic microtubule polymerisation. This was accompanied by the upregulation and downregulation of several proteins. The differentially expressed proteins pyruvate kinase, enolase-1 and glucose dehydrogenase are part of the glycolytic metabolism and their regulation correlates well with the cell cycle arrest in G_2_/M after LASP-1 silencing. Furthermore, pyruvate kinase and glucose dehydrogenase have been suspected to be highly important for tumour cell metabolism (Altenberg and Greulich, 2004). Pyruvat kinase is one of the proteins to be upregulated in cancer cells gaining energy by means of aerobic glycolysis, which is a characteristic of a number of cancer entities (Gatenby and Gillies, 2004). In addition, pyruvat kinase has been identified as a proteomic marker of cancer progression in breast cancer (Isidoro *et al*, 2005). The glycolytic enzyme enolase-1 as well as HSP27, two additional proteins identified in the 2D-gel experiments, are associated with high metastatic activity in breast cancer cells (Espana *et al*, 2005; Zhang *et al*, 2005).

14-3-3, found to be upregulated after LASP-1 depletion in ovarian cancer cells, has been implicated in cell cycle deregulation. The 14-3-3 proteins are a family of highly conserved DNA-binding proteins, which associate with the centrosomes during mitosis and are inhibitors of G_2_/M progression at the mitotic and G_2_ cell cycle checkpoint (Pietromonaco *et al*, 1996; Wang and Shakes, 1996; Hermeking *et al*, 1997; Peng *et al*, 1997; Alvarez *et al*, 2002). Overexpression of 14-3-3 led to cell cycle arrest in cell culture models (Tzivion *et al*, 2006) and, therefore, might contribute to the observed G_2_ arrest in ovarian cancer cells lacking LASP-1.

Heat-shock proteins are molecular chaperons and are induced during cellular stress. Upregulation of HSP27 after LASP-1 silencing correlates well with increased survival by inhibiting key effectors of the apoptotic pathway (Concannon *et al*, 2003).

So far, the identified proteins are regulated in response to cell cycle arrest, but do not substitute for LASP-1 after silencing.

Recently, several LASP-1-binding partners have been identified. Along with zyxin (Li *et al*, 2004) and actin (Schreiber *et al*, 1998), LASP-1 interacts with Krp1 (Spence *et al*, 2006), palladin (Rachlin and Otey 2006), lipoma-preferred partner (LPP) and VASP (Keicher *et al*, 2004), which all can influence actin filament dynamics and pseudopodial elongation. In the case of palladin, LPP and zyxin, the binding occurs between the C-terminal SH3 domain of LASP-1 and the N-terminal proline-rich domains of these proteins, whereas in the case of Krp1, binding is observed between the nebulin-like repeats of LASP-1 and the N-terminal BTB/POZ domain of Krp1. The interaction of LASP-1 and Krp1 is crucial for pseudopodial elongation in fibroblasts in absence of fibronectin and results in their colocalisation with F-actin at the tips of extending pseudopodia (Spence *et al*, 2006).

Zyxin is localised primarily at focal adhesion plaques and plays a central role in actin filament polymerisation in mammalian cells (Beckerle, 1997).

Silencing of zyxin in HeLa cells resulted in significantly reduced actin stress fibres (Griffith *et al*, 2005), whereas under cyclic stretch zyxin only dissociated from focal contacts and accumulated in the nucleus, without affecting vinculin or actin filaments (Cattaruzza *et al*, 2004). Recent data show that in genetically zyxin-deficient fibroblasts, focal adhesions are depleted from Mena and VASP, and that cells lacking zyxin display deficits in actin cytoskeleton remodelling (Hoffman *et al*, 2006). In our immunflourescence experiments with LASP-1-deficient SKOV-3 cells, we observed a diffuse cytoplasmic localiation of zyxin without protein loss and without changes in neither vinculin distribution nor actin stress fibre organisation, emphasising the importance of LASP-1 for binding and recruiting zyxin to focal adhesions.

The loss of zyxin at the sites of focal contacts without changing cellular zyxin protein levels is not restricted to cancer cells, but was also observed in human umbilical vein endothelial cells (Grunewald *et al*, 2006). Interestingly, in these cells, zyxin could still be detected along the actin stress fibres, indicating the potential existence of another zyxin-recruiting protein along actin stress fibres since earlier results detected LASP-1 only in the focal adhesion plaques (Chew *et al*, 2002; Butt *et al*, 2003).

In our zyxin knock-down experiments, neither changes in LASP-1 localisation, actin cytoskeleton, microtubule polymerisation nor vinculin distribution were detectable suggesting that zyxin does not change focal adhesion morphology. This is concordant with the fact that genetically zyxin-deficient fibroblasts show even enhanced adhesion to surface and increased integrin expression (Hoffman *et al*, 2006). In synopsis, our LASP-1 and zyxin silencing studies have demonstrated that LASP-1 is necessary for recruiting zyxin to focal contacts.

The decreased cell motility after LASP-1 silencing can be explained by the functional loss of zyxin as a scaffolding protein that facilitates the formation of molecular complexes to promote site-specific actin assembly required for cell migration. This is in agreement with previous findings using a nongenetic approach and injecting a peptide derived from the N-terminus of zyxin to displace zyxin from its normal subcellular location thus leading to reduced cell migration (Drees *et al*, 1999). On the other hand, the knock-down of zyxin in SKOV-3 cells had no influence on cell migration while genetically zyxin-deficient fibroblasts display enhanced migration (Hoffman *et al*, 2006). These contrary effects have not been fully elucidated yet.

Recent work has shown that zyxin also shuttles through the nucleus – most likely by association with other LIM proteins – and may regulate gene transcription (Nix *et al*, 2001; Wang and Gilmore, 2003; Kadrmas and Beckerle, 2004). During mitosis, a fraction of zyxin becomes associated with the tumour suppressor h-warts at the mitotic apparatus (Hirota *et al*, 2000). h-warts is a key player in mitosis in mammalian cells and loss of its function disrupts normal cell cycle regulation, possibly leading to tumour development (Iida *et al*, 2004). In SKOV-3 cells transfected with LASP-1 siRNA, zyxin has been shown to dissociate from focal adhesion plaques and to distribute diffusely into the cytoplasm. It is, therefore, likely that part of zyxin enters the nucleus, binds to h-warts and leads to G_2_ cell cycle arrest and inhibition of proliferation as observed after LASP-1 silencing.

Interestingly, in Ewing tumour cells, zyxin is only expressed at very low levels and remains diffusely distributed throughout the cytoplasm instead of concentrating in actin-rich dynamic structures. Zyxin gene transfer into EWS-FLI1-transformed fibroblasts elicits reconstitution of zyxin-rich focal adhesions and leads to decreased cell motility and inhibition of anchorage independent tumour growth, indicating that zyxin has tumour suppressor activity in these cells (Amsellem *et al*, 2005).

Similar to findings in human breast cancer (Grunewald *et al*, 2006), our immunofluorescence experiments have shown that absence of LASP-1 in focal contacts dramatically influences zyxin distribution. In reverse, tumour cells, that are overexpressing LASP-1, could functionally inhibit zyxin from shuttling into the nucleus and acting as a tumour suppressor through increased recruiting of zyxin to focal contacts by LASP-1. In summary, our observations suggest an expanded role for LASP-1 in proliferation and cancer cell migration. Further studies will define the potential of LASP-1 as an independent marker for diagnosis of cancer, as well as a marker for prognosis of this disease.
